# Blood pressure and renal outcomes after renal artery aneurysm intervention: Single-center experience and review of literature

**DOI:** 10.3389/fcvm.2023.1127154

**Published:** 2023-04-21

**Authors:** Siting Li, Fangda Li, Zhili Liu, Rong Zeng, Wei Ye, Jiang Shao, Yuehong Zheng

**Affiliations:** ^1^ Department of Vascular Surgery, Peking Union Medical College Hospital, Chinese Academy of Medical Sciences and Peking Union Medical College, Beijing, China; ^2^ Department of State Key Laboratory of Complex Severe and Rare Diseases, Peking Union Medical College Hospital, Chinese Academy of Medical Science and Peking Union Medical College, Beijing, China

**Keywords:** renal artery aneurysm, blood pressure, renal outcome, endovascular repair, open surgery

## Abstract

**Objective:**

To explore the results of hypertension improvement and renal function preservation after renal artery aneurysm (RAA) repair.

**Methods:**

This study retrospectively analyzed the change in blood pressure (BP) and renal outcomes of 59 RAA patients throughout either open or endovascular operations and follow-up at a large center. Patients were grouped according to the difference in their BP at the last follow-up vs. their baseline value. Logistic regression was conducted to explore risk factors for perioperative BP relief and long-term hypertension reonset. Previous studies of RAA with records of BP, blood creatinine level, or GFR/eGFR results are reviewed.

**Results:**

Hypertension was observed in 62.7% (37/59) of the patients included. Postoperative BP declined from 132.20 ± 16.46/79.92 ± 9.64 mmHg to 122.41 ± 11.17/71.10 ± 9.82 mmHg, while eGFR changed from 108.17 ± 24.73 to 98.92 ± 23.87 ml/min/1.73 m^2^. The median follow-up was 854 [IQR: 1,405] days. Both open and endovascular techniques significantly relieved hypertension and did not impair renal function much. Lower preoperative systolic BP (SBP) was significantly associated with hypertension relief (OR = 0.83, 95% CI: 0.70–0.99). Among patients with normal BP after the operation, higher postoperative SBP was significantly associated with new-onset hypertension (OR = 1.14, 95% CI: 1.01–1.29). Literature review indicated that renal function usually remained normal at follow-up, whereas relief of hypertension varied.

**Conclusion:**

Patients with lower preoperative SBP were likely to benefit more from the operation, while higher postoperative SBP indicated a higher chance of hypertension reonset. Creatinine level and eGFR generally remained stable regardless of operation type.

## Introduction

Renal artery aneurysm (RAA) is a relatively rare condition that entails high risk if ruptured (1). Around two-thirds of RAA patients have concomitant hypertension, and refractory high blood pressure (BP) is also an indication for surgical intervention (2). The safety and efficacy of both open surgery and endovascular approaches have been summarized at various centers (3–7). Although relief of high BP following repair has been reported, hypertension reonset is common (4, 8). The prevalence of resistant and true resistant hypertension (9) for RAA is not known, and the mechanism underlying BP changes has not been fully elucidated (5). Despite the generally good baseline conditions of most RAA patients, alteration of creatinine or other indicators for renal function has not been thoroughly discussed (2).

In this study, we retrospectively analyzed the changes in BP and renal outcomes in 59 RAA patients during either open or endovascular operation and throughout follow-up at our institution. Previous studies that had recorded the detailed values of BP, blood creatinine, or glomerular filtration rate (GFR) in patients undergoing RAA open or endovascular operation are reviewed. Multivariable logistic regression was conducted to identify the predictive factors of perioperative BP relief and long-term hypertension reonset.

## Methods

### Study population and design

From 2009 to 2021, patients diagnosed with RAA who underwent therapy at the Department of Vascular Surgery, Peking Union Medical College Hospital (PUMCH) were consecutively included in the study. Patients without a detailed record of BP levels were excluded. Computed tomography angiography (CTA), magnetic resonance angiography (MRA), or digital subtraction angiography (DSA) was utilized for RAA diagnosis and evaluation. All participating subjects provided written informed consent, and the study was approved by the institutional review board.

Baseline characteristics, perioperative results, and follow-up information were retrospectively collected and analyzed. Indications for intervention included aneurysm diameter >2 cm and symptomatic RAA (rupture, hematuria, refractory hypertension not controlled by ≥3 antihypertensive agents of different classes, and flank/abdominal/chest pain excluded of other reasons). Both endovascular and open techniques were applied for treatment.

Regarding treatment strategies, coil embolization, as a minimally invasive endovascular procedure with good efficacy and safety, was the first-line treatment if suitable. Stent-assisted coiling (SAC) or simple stent implantation was applied for complicated lesions. For those who were unsuitable for endovascular therapy or too young, open surgery including aneurysmoplasty, resection and primary anastomosis, tailoring, ex vivo repair, and resection and graft reconstruction with either the saphenous vein or a prosthetic graft, was selectively conducted. Detailed procedures can be found in previous literature and in supplemental Table 1 (1, 3, 10–12).

**Table 1:** Disease parameters in all patients and comparison among different hypertension groups.

	All (*n* = 59)	Group 1. Always normal (*n* = 18)	Group 2. Complete relief (*n* = 6)	Group 3. Partial relief (*n* = 18)	Group 4. No relief (*n* = 4)	Group 5. Relief and new onset (*n* = 9)	Group 6. New onset (*n* = 4)	*p*
Gender/female	36 (61.0)	11 (61.1)	2 (33.3)	11 (61.1)	2 (50.0)	7 (77.8)	3 (75.0)	0.665
Age, year	48.88 ± 13.57	44.44 ± 12.36	48.67 ± 15.38	52.76 ± 16.27	58.25 ± 6.65	47.00 ± 12.22	47.25 ± 10.50	0.327
BMI, kg/m^2^	24.80 ± 3.53	24.56 ± 2.59	24.76 ± 2.51	23.58 ± 2.53	29.15 ± 5.20	24.89 ± 3.01	23.08 ± 1.32	0.258
Smoking, *n* (%)	9 (15.3)	3 (16.7)	2 (33.3)	2 (11.1)	2 (50.0)	0	0	0.167
diabetes, *n* (%)	15 (25.4)	11 (61.1)	2 (33.3)	1 (5.6)	1 (25.0)	0	0	0.178
dyslipidemia, *n* (%)	12 (20.3)	2 (11.1)	1 (16.7)	5 (27.8)	2 (50.0)	2 (22.2)	0	0.474
Aneurysm diameter, cm [IQR]	2.00 [1.62]	2.31 [1.83]	2.00 [1.10]	2.22 [1.27]	1.25 [1.05]	2.89 [2.60]	2.55 [1.45]	0.284
symptomatic, *n* (%)	22 (37.3)	4 (22.2)	3 (50.0)	5 (27.8)	3 (75.0)	5 (55.6)	2 (50.0)	0.206
Endovascular repair, *n* (%)	45 (76.3)	14 (77.8)	4 (66.7)	14 (77.8)	4 (100.0)	6 (66.7)	3 (75.0)	0.866
Pre-op number of anti-hypertensive drugs	0.89 ± 0.96	0	1.33 ± 1.21	1.31 ± 0.75	1.33 ± 0.58	1.40 ± 0.55	0	**<0.001**
History of hypertension, *n* (%)	37 (62.7)	0	6 (100.0)	18 (100.0)	4 (100.0)	9 (100.0)	0	**<0.001**
Average CKD grade	1.15 ± 0.36	1.19 ± 0.40	1.00 ± 0.00	1.06 ± 0.25	1.25 ± 0.50	1.25 ± 0.46	1.33 ± 0.58	0.385
Pre-op sys, mmHg	132.20 ± 16.46	116.08 ± 11.02	138.67 ± 21.71	143.15 ± 11.97	152.00 ± 16.09	137.00 ± 11.85	126.33 ± 17.21	**<0.001**
Pre-op dia, mmHg	79.92 ± 9.64	73.83 ± 9.50	90.5 ± 3.79	83.08 ± 6.75	82.67 ± 3.79	72.80 ± 9.42	80.33 ± 11.93	**0.005**
Post-op sys, mmHg	122.41 ± 11.17	115.22 ± 9.67	123.17 ± 16.39	124.11 ± 8.51	134.50 ± 10.79	128.00 ± 8.57	121.25 ± 9.61	**0.007**
Post-op dia, mmHg	71.10 ± 9.82	67.67 ± 7.49	78.17 ± 17.29	70.67 ± 8.58	76.75 ± 6.30	74.11 ± 9.64	65.50 ± 7.85	0.149
Pre-op eGFR, ml/min/1.73 m^2^	108.17 ± 24.73	107.86 ± 14.86	102.86 ± 11.47	92.04 ± 26.38	110.38 ± 19.14	109.01 ± 34.21	105.19 ± 19.88	0.789
Post-op eGFR, ml/min/1.73 m^2^	98.92 ± 23.87	108.20 ± 21.13	101.43 ± 14.35	88.55 ± 29.16	101.78 ± 23.29	97.82 ± 27.46	99.78 ± 22.12	0.825
Long-term number of types of anti-platelet drugs	0.53 ± 0.63	0.58 ± 0.52	0.67 ± 0.82	0.85 ± 0.69	0.33 ± 0.58	0.20 ± 0.45	0.33 ± 0.58	0.840

BMI, body mass index; eGFR, estimated glomerular filtration rate. IQR: interquartile range. CKD: chronic kidney disease. Significant *p* value was marked in bold.

### Hypertension and renal function evaluation

Hypertension was defined as arterial systolic (SBP) or diastolic BP (DBP) levels of three or more clinical measurements above 140/90 mmHg or taking antihypertensive drugs on admission (13). BP levels were collected upon admission, on the next day after operation, and at follow-up. Patients were grouped into 6 groups according to the difference in their BP results at the last follow-up vs. baseline value:

(1) Always normal patients had normal BP throughout the study.
(2) Complete relief of hypertension was defined as SBP and DBP at three or more clinical measurements below 140/90 mmHg without anti-hypertensive drugs.
(3) Partial relief was defined as SBP and/or DBP below 140/90 mmHg while taking the same number or reduced number of medications, or a reduction in DBP by at least 15-mm Hg while on the same or reduced number of medications.
(4) No relief was defined as no change or inability to meet the above criteria.
(5) New onset referred to patients with newly-diagnosed hypertension at follow-up.
(6) Relief and new onset was defined as BP relief at discharge but had new-onset hypertension at follow-up.

Chronic kidney disease was graded according to the National Kidney Foundation Kidney Disease Outcomes Quality Initiative classification (14). Estimated GFR (eGFR) was calculated with the Cockcroft-Gault equation, impaired renal function being defined as a 30% reduction in eGFR.

### Follow up

Thirty-day and long-term follow-up data were recorded and explored. Technical primary success was defined as the successful introduction and deployment of the device in the absence of surgical conversion or any complication. Color Doppler, CTA, or angiography was applied to evaluate arterial patency, which was defined as evidence of blood flow within the target renal artery or bypass and perfusion in the respective renal parenchyma. Laboratory tests, including creatinine (µmol/L), white blood cell count (WBC, 109/L), hemoglobin (HGB, g/L), platelet count (PLT 109/L), and D-dimer (mg/L), as well as ultrasounds of the kidney or renal artery, were done at least once preoperatively, postoperatively before discharge, and at three months, six months, and one year or yearly after discharge.

### Logistic regression for perioperative BP relief and long-term hypertension re-onset

Logistic regression of hypertension relief was done between group 2, group 3, group 4, and group 5 patients, who had hypertension before the operation. Patients were divided into complete relief (group 2 and group 5) and incomplete relief (group 3 and group 4). Ordinal regression was first performed between the complete relief group and the incomplete relief group to identify factors with significant associations, which were then incorporated into the multivariate logistic regression model.

Logistic regression of new-onset hypertension was run between group 1, group 2, group 5, and group 6 patients who had normal BP after the operation. Patients were divided into new-onset (groups 5 and 6) and no onset (groups 1 and 2) groups. Similar factors as from the ordinal regression model were incorporated.

### Literature research and review

Literature searches in English and Chinese were performed in PubMed, EMBASE and Scopus using the keyword “renal artery aneurysm”. Studies that reported the detailed preoperative, postoperative, follow-up, or changed values of BP, blood creatinine level, or GFR/eGFR level in patients who underwent open or endovascular operation for RAA were included. Single-case reports were excluded. Two authors (SL and FL) reviewed the original articles and identified 10 papers on BP and 16 papers on renal function published from 2001 to 2022.

### Statistical analysis

Statistical analysis was performed using SPSS 26.0 software (IBM Corp, Armonk, NY). Continuous variables were checked for normality and are reported as median and interquartile range (IQR) or mean ± standard deviation. Categorical variables are presented as frequencies and were compared by the *χ*^2^ test or Fisher's exact test, as appropriate. The Wilcoxon test or the Kruskal‒Wallis and Friedman tests were employed for cross- or within-group comparisons, respectively. *p* < 0.05 was considered significant.

## Results

### Patient demographics

Table 1 showed the basic characteristics of all patients and for each group with different BP conditions. Of the 59 subjects included, 61.0% (36) were female. The average age was 48.88 ± 13.57 years. Smoking, diabetes, and dyslipidemia were present in 15.3% (9), 25.4% (15), and 20.3% (12) of all patients, respectively. The median diameter of the aneurysm was 2.00 [IQR 1.62] cm. Regarding aneurysm position, most of the patients had aneurysms located at the first bifurcation (25, 42.4%), followed by 23 (22.0%) at the first-order branch, 13 (12.0%) at the main renal artery, 5 (8.50%) at both the first-order branch and the main renal artery, and 3 (5.1%) at intralobular branches. Hypertension was observed in 62.7% (37) of all patients, and 37.3% (22) complained of pain. One patient had concomitant renal artery stenosis. The average chronic kidney disease grade was 1.15 ± 0.36. Endovascular repair was performed in 45 (76.3%) patients. Postoperatively, average BP declined from 132.20 ± 16.46/79.92 ± 9.64 mmHg to 122.41 ± 11.17/71.10 ± 9.82 mmHg, while eGFR changed from 108.17 ± 24.73 to 98.92 ± 23.87 ml/min/1.73 m^2^.

### Follow-up results

The median follow-up was 64.5 [IQR: 65.2] and 25.1 [IQR: 33.1] months for patients receiving endovascular repair and open surgery, respectively (Supplementary Table S2). No significant difference in primary technical success, postoperative complications, reocclusion, or reintervention rate for open and endovascular repair was observed. Two patients in each group had occluded renal arteries but were treated conservatively because of preserved renal function. Five (11.5%) of the patients receiving endovascular repair underwent a second endovascular operation because of unsatisfactory aneurysm embolization. One patient received coiling 9 months after undergoing the tailoring technique due to unsatisfactory resolution of the aneurysm, and 1 received ballooning and stent implantation for anastomotic stenosis after ex vivo repairs and autotransplantation.

An average number of types of 0.53 ± 0.63 antiplatelet drugs (aspirin or clopidogrel) were prescribed for patients at the last follow-up (Table 1). Laboratory results at different time points after RAA repair are illustrated in Supplementary Figure S1. WBC, HGB, PLT, and D-dimer fluctuated briefly after the operation but returned to baseline at both the short-term and long-term examinations (Supplementary Table S1). No significant difference was observed in the above values between the open and endovascular groups in the long run (Supplementary Table S2).

### BP and renal function results

All patients were divided into 6 groups according to their BP pattern (Table 1). Eighteen patients had constant normal BP (group 1), 6 complete relief of hypertension (group 2), 18 partial relief (group 3), 4 no relief (group 4), 9 hypertension relief but long-term new-onset (group 5), and 4 new-onset hypertension only at the follow-up test (group 6). Among patients with partial relief, 4 (22.2%) reduced their number of medications. Patients with no relief took the same number or more medications. Kruskal-Wallis test among all subgroups suggested no significant difference in comorbidity between patients with different BP statuses, but significant differences were observed in the number of antihypertensive medicines (*p* < 0.001), prevalence of hypertension history (*p* < 0.001), preoperative SBP and DBP values (*p* < 0.001 and =0.005), and postoperative SBP (*p* = 0.007).

BP and renal function alterations were also explored and compared between patients who underwent open or endovascular repair (Table 2). There was no significant difference between the two approaches perioperatively or in the long run. Both SBP and DBP decreased significantly after the operation. Patients receiving endovascular repair took significantly fewer antihypertensive medicines. Regarding renal outcomes, the endovascular group had a transient increase in creatinine (*p* = 0.047), but no significant difference was observed in the long-term results.

**Table 2:** Change of blood pressure and renal function for RAA patients.

	Open (*n* = 14)	*p* vs. baseline	Endovascular (*n* = 45)	*p* vs. baseline	*p*
Pre-op SBP (mmHg)	133.43 ± 20.32		131.82 ± 15.31		0.824
Post-op SBP	118.43 ± 13.20	**0.011**	123.64 ± 13.20	**0.001**	0.349
Pre-op DBP (mmHg)	79.86 ± 10.75		79.93 ± 9.40		0.796
Post-op DBP	69.93 ± 7.29	**0.028**	71.47 ± 10.53	**<0.001**	0.789
Pre-op number of medicine	0.92 ± 0.95		0.82 ± 0.86		0.847
Post-op number of medicine	0.77 ± 0.73	0.317	0.61 ± 0.69	**0.012**	0.667
Follow-up number of medicine	0.62 ± 0.77	0.180	0.64 ± 0.68	**0.018**	0.814
Pre-op creatinine (µmol/L)	68.77 ± 12.54		63.07 ± 12.21		0.304
Post-op creatinine	72.31 ± 15.42	0.109	65.61 ± 11.33	**0.047**	0.319
Follow-up creatinine	73.29 ± 15.41	0.735	71.79 ± 17.32	0.083	0.743

SBP, systole blood pressure; DBP, diastole blood pressure. Significant *p* value was marked in bold.

### Risk factors for perioperative BP relief and long-term hypertension reonset

To further investigate the risk factors for perioperative BP relief and long-term hypertension reonset, logistic regression was conducted in two different sets of groups (Table 3). Higher preoperative low-density lipoprotein (LDL) level was significantly associated with hypertension relief after the operation (OR = 65.55, 95% CI: 1.12–3,833.03). Lower preoperative SBP was significantly associated with hypertension relief (OR = 0.83, 95% CI: 0.70–0.99). Among patients who had normal BP values after the operation, higher postoperative SBP was significantly associated with new-onset hypertension (OR = 1.14, 95% CI: 1.01–1.29).

**Table 3:** Predictors for hypertension relief after operation and new onset at follow-up.

	Hypertension relief^a^		Hypertension new onset^b^	
	OR	*p*	OR	*p*
Age (year)	0.92 (0.81–1.05)	0.228	1.07 (0.97–1.18)	0.175
Aneurysm diameter (cm)	1.32 (0.54–3.27)	0.544	1.70 (0.69–4.20)	0.249
Pre-op creatinine (µmol/L)	1.13 (0.98–1.30)	0.102	0.91 (0.83–1.01)	0.064
Pre-op LDL (mmol/L)	65.55 (1.12–3,833.03)	**0.044**	4.01 (0.56–28.73)	0.167
Pre-op SBP (mmHg)	0.83 (0.70–0.99)	**0.042**	NA	NA
Post-op SBP (mmHg)	NA	NA	1.14 (1.01–1.29)	**0.038**

LDL, low density lipoprotein; OR, odds ratio; NA, not available. Significant *p* value was marked in bold.

^a^ Logistic regression of hypertension relief was calculated among groups 2,3, 4, and 5 patients who had hypertension before operation. Patients were divided into complete relief (group 2 and 5) and not complete relief (group 3 and 4).

^b^ Logistic regression of hypertension new-onset was calculated among groups 1, 2, 5, and 6 patients who had normal BP after the operation. Patients were divided into new-onset (group 5 and 6) and no on-set (group 1 and 2).

## Discussion

The present study demonstrated good results from our twelve-year experience with RAA repair. No significant difference was found in the perioperative laboratory parameters between the open and endovascular groups. Patients receiving open surgery had a transient increase in D-dimer and PLT levels, but their long-term laboratory tests returned to normal. A significantly higher rate of reintervention was observed among patients with new-onset hypertension than for those with other BP statuses. Since RAA patients were generally younger and healthier than other patients in the vascular surgery ward, hypertension improvement and renal function preservation were crucial to reduce morbidity and minimize any potential risk for cardiovascular events in the long run.

The mechanism of hypertension in RAA remains unclear. Concurrent RAS or compression might play a part, while microembolization in the distal renal parenchyma and hemodynamic changes from turbulent blood flow within the aneurysm could also contribute (2). The resolution of these conditions by operation may improve the hypertension (15). Our study suggested that despite an overall significant decrease in both SBP and DBP as well as in the number of anti-hypertension medicines taken postoperatively, it must be noted that baseline BP conditions, the extent of hypertension relief, and hypertension occurrence varied between individuals. Some studies have suggested distinct results between patients with renovascular hypertension and those without evidence of stenosis, but this difference was not observed in our study (4, 16, 17).

Concerning renal function, most RAA patients in this study had normal baseline levels of creatinine and eGFR. During operation, the introduction of nephrotoxic iodinated contrast medium, embolization of a part of the renal parenchyma, failure of artery reconstruction, and clamping of blood supply may all increase the risk of renal impairment (18). Nevertheless, our study showed that creatinine and eGFR remained stable over the long-term follow-up regardless of operation type.

To further explore the problem of RAA and BP or renal function, we reviewed the results of BP and indicators of renal function reported in previous studies. Although expertise in RAA repair has accumulated rapidly over the last decade, few studies have reported on the exact values of these parameters. Ten papers that reported the value of BP for RAA were included (Table 4) (3, 7, 8, 10, 19–23). Of all 367 patients included, 345 received open therapy, while 50 underwent endovascular repair. The preoperative average SBP value was 146.90 mmHg. Two of 4 studies that recorded both preoperative and postoperative BP found a significant reduction, and 6 of 7 studies that recorded preoperative and follow-up results found a significant reduction. Regarding the number of antihypertensive medicines, follow-up results from 3 studies all reported a decrease. Sixteen papers concerning renal function were included (Table 5) (7, 8, 10, 18–21, 23–29). In total, 277 and 162 patients received open or endovascular repair, respectively. Twelve studies recorded both preoperative and postoperative creatinine levels, of which 2 suggested a significant increase and 1 a significant decrease. One of the 5 studies reporting preoperative and follow-up results observed a significant increase in creatinine. Only 1 study showed a significant increase in GFR transiently after the operation, but not at follow-up. In general, in line with our study, no significant deterioration of renal function was observed except for transient fluctuation. A significant decrease in SBP or DBP or antihypertensive drug number has been reported in both the short term and long term in some conditions. In some other cases, hypertension was not fully relieved after the operation, and medicines were required for BP control. It should also be noted that most of these patients received open surgical repair for RAA, which may not represent the whole picture given the rise in popularity of the endovascular approach.

**Table 4:** Previous reports on hypertension status for RAAs.

Ref.	Pts	Interve-ntion	Technical success	Follow-up/m	Percentage of high BP	Pre-op BP, SBP/DBP	Post-op BP, SBP/DBP	*p* vs. baseline	Follow-up BP, SBP/DBP	*p* vs. baseline	Pre-op num. of medicine	Post-op num. of medicine	*p* vs. baseline	Follow-up num. of medicine	*p* vs. baseline
Henke et al.	47	Open	91.7%	94	73%	153.13/91.96	NA	NA	138.02/78.81	**<0.004**	1.78	NA	NA	1.00	**<0.001**
Robinson et al.	24	Open	88.5%	NA	100%	142 ± 18/86 ± 14	130 ± 15/78 ± 10	**0.007/0.01**	NA	NA	2.6 ± 1.5*	1.1 ± 0.8*	**0.0128**	NA	NA
Duprey et al.	67	Open	92.0%	96	82%	143 ± 24/82 ± 16	NA	NA	127 ± 16/72 ± 10	**<0.05**	2 ± 1	NA	NA	0.94 ± 0.13	**<0.05**
Steuer et al.	9	Open	100%	12	66.6%	140 (115–170)/90 (80–100)	140 (120–155)/	0.44	150 (140–170)/	0.22	1.11	1.0 (0–4)	0.32	NA	NA
Jayet et al.	21	Open	100%	24	71.4%	134 ± 21/74 ± 10	123 ± 12/73 ± 10	**SBP: <0.05**	122.3/	**SBP: 0.047**	1.4 ± 1.2	NA	NA	NA	NA
Chandra et al.	9	Open	100%	NA	78%	138 ± 20/	141 ± 19/	0.64	NA	NA	2.7	1.6	**0.03**	NA	NA
English et al.	62	Open	99%	48	89%	171 ± 35/95 ± 19	NA	NA	134 ± 20/82 ± 10	**0.0001**	2.2	NA	NA	1.5	**0.02**
Duran et al.	80	Open	90.91%	60.7	70%	138.3 ± 18.5/	NA	NA	128.3 ± 13.1/	**SBP: <0.001**	NA	NA	NA	NA	NA
Wei et al.	28	Endo	100%	19	61%	136 ± 21/82 ± 9	NA	NA	125 ± 8/70 ± 4	**<0.05**	NA	NA	NA	NA	NA
Tsilimparis et al.	20	Open	95.0%	25	75%	NA	NA	NA	NA	NA	1.6 ± 1.1	NA	NA	2 ± 1.5	NA
	22	Endo	95.5%	27	30%	NA	NA	NA	NA	NA	1.5 ± 1.4	NA	NA	1.7 ± 1.2	NA

Blood pressure was listed with the unit of mmHg.

* In 11 patients with severe hypertension. NA, Not available. Significant *p* value was marked in bold.

**Table 5:** Previous reports on renal function for RAAs.

Ref.	Pts	Intervent-ion	Technical success	Follow-up/m	Pre-op creatinine	Post-op creatinine	*p* vs. baseline	Follow-up creatinine	*p* vs. baseline	Pre-op GFR or eGFR	Post-op GFR or eGFR	*p* vs. baseline	Follow-up GFR or eGFR	*p* vs. baseline
Robinson et al.	24	Open	88.5%	99	82.21 ± 26.52	93.70 ± 35.36	0.11	NA	NA	NA	NA	NA	NA	NA
Duprey et al.	67	Open	92.0%	96	NA	NA	NA	NA	NA	93 ± 29*	94 ± 33*	>.05	86 ± 26*	0.06
Steuer et al.	9	Open	100%	12	84	125	0.22	85	0.07	88	NA	NA	NA	NA
Jayet et al.	24	Open	100%	24	67 ± 13	57 ± 12	**<0.05**	63 ± 11	>0.05	93 ± 49	125 ± 59	**0.02**	95 ± 35	0.56
Chandra et al.	9	Open	100%	11.9	NA	NA	NA	NA	NA	81.2	NA	NA	79.2	0.81
Duran et al.	80	Open	90.91%	60.7	75.14 ± 18.56	79.56 ± 26.52	0.164	NA	NA	NA	NA	NA	NA	NA
Chung et al.	9	Endo	100%	29.8	72 (48–92)	79 (55–105)	**0.03**	62–98	NS.	NA	NA	NA	NA	NA
Tang et al.	32	Endo	100%	NA	76.5	83.0	**0.012**	NA	NA	NA	NA	NA	NA	NA
Jibiki et al.	16	Open	93.75%	NA	68.95 ± 17.68	66.30 ± 26.52	NS.	NA	NA	71 ± 16	78 ± 28	NS.	NA	NA
Zhang et al.	9	Endo	100%	NA	63.4	65.0	0.593	NA	NA	131.4	119.7	0.286	NA	NA
Abreu et al.	9	Endo	88.9%	NA	79.56 ± 17.68	80.44 ± 38.90	NS.	NA	NA	80 (66–97)	77 (57–95)	NS.	NA	NA
Kawase et al.	14	Open	100%	NA	61.88 ± 12.38	65.42 ± 13.26	NS.	NA	NA	NA	NA	NA	NA	NA
Wei et al.	28	Endo	100%	19	81.2 ± 30.6	NA	NA	60.82 ± 5.7	<0.01	88.8 ± 25.9	NA	NA	105.9 ± 15.9	**<0.01**
Ma et al.	40	Endo	97.5%	11.4	66.97 ± 23.13	75.50 ± 25.58	**0.0003**	NA	NA	NA	NA	NA	NA	NA
Laurin et al.	14	Open	88.9%		73.4 ± 17.6	70.7 ± 17.7	NS.	85.1 ± 18.0	NA	93.2 ± 15.8	93.9 ± 12.9	NS.	70.8 ± 12.2	0.09
	13	Endo	100%		66.1 ± 14.1	64.1 ± 15.4	NS.	73.7 ± 11.9	NA	91.0 ± 18.9	97.0 ± 20.4	NS.	81.9 ± 23.2	
Tsilimparis et al.	20	Open	95.0%	25	NA	NA	NA	NA	NA	NA	NA	NA	*Δ*: −7 ± 27	NS.
	22	Endo	95.5%	27	NA	NA	NA	NA	NA	NA	NA	NA	*Δ*: −11 ± 23	NS.

Creatinine was listed with the unit of µmol/L; GFR or eGFR was listed with the unit of ml/min/1.73 m^2^.

* Calculated by MDRD clearance. NS., Not significant; NA, Not available; Ref., reference; Pts, patients; Endo, endovascular. Significant *p* value was marked in bold.

Since controversial results exist, we sought to explore the underlying factors that may contribute to BP alteration using logistic regression models. As for hypertension relief after the operation, we found that patients with lower SBP and higher blood LDL levels preoperatively achieve better outcomes, which could provide predictive value for BP alteration after the operation. Several previous studies concerning BP response after operation for RAS have identified the level of SBP or DBP as a positive predictor of hypertension relief (30, 31). However, as many of these patients have renovascular hypertension, renal artery stenting could resolve this condition and directly relieve the hypertension. Only one patient in our study had a clear concomitant RAS on imaging. Hypertension in RAA patients, on the other hand, may have more complicated origins and cannot be easily cured by renal artery revascularization. As for LDL levels, little evidence of their effect has been found. On account of the wide CI range of this factor in our patients, we assumed that our LDL findings could be a false positive resulting from extreme individual values. Additionally, we observed a trend (though not significant) that greater aneurysm diameter, greater preoperative creatinine, and earlier age were positively associated with the extent of BP relief, which suggested that a combination of more underlying factors may contribute to hypertension in RAA.

Patients with higher postoperative SBP, though within the normal range, might still be at higher risk of new-onset hypertension. For these patients, renal aneurysm may not be the primary factor causing the hypertension. Thus, the operation could not fully stop the progression of hypertension. A previous study also suggested that a longer duration of hypertension may bring an unfavorable outcome (32). Since hypertension was associated with a higher chance of reintervention, postoperative BP monitoring would be more valuable for these patients.

This study has some limitations. Besides blood creatinine and eGFR, a more thorough evaluation of renal function, such as urine tests and CTA, was not normally conducted at follow-up, and ambulatory BP monitoring was not done. Subgroup analysis was hampered by the small subject numbers, making it difficult to reach robust conclusions. The activity of the renin-angiotensin system (RAAS) was not routinely tested, so the underlying pathophysiology and etiology of BP change might not be fully explained. More evidence is required to set the optimal RAA diameter threshold for surgical intervention. Nevertheless, through a literature review, we discussed the nature of BP and renal function alteration after RAA operation, which could assist in prognostic prediction for these patients. Larger multicenter studies are necessary to validate our findings.

## Conclusion

Both open and endovascular techniques for RAA repair demonstrated significant relief of hypertension and little impairment of renal function, yet baseline BP conditions and the extent of hypertension relief or long-term occurrence varied. Patients with lower preoperative SBP were likely to benefit more from the operation, while higher postoperative SBP indicated a higher chance of long-term hypertension reonset. Creatinine level and eGFR generally remained stable regardless of operation type.

## Data Availability

The data that support the findings of this study are available from the corresponding author upon reasonable request.
