# Supplementary material for: Blood pressure and renal outcomes after renal artery aneurysm intervention: Single-center experience and review of literature
Source: Front Cardiovasc Med. 2023 Apr 21;10:1127154. doi: 10.3389/fcvm.2023.1127154 (PMC10160466; doi:10.3389/fcvm.2023.1127154)
Supplement: Supplementary file 1 [file Datasheet1.docx]

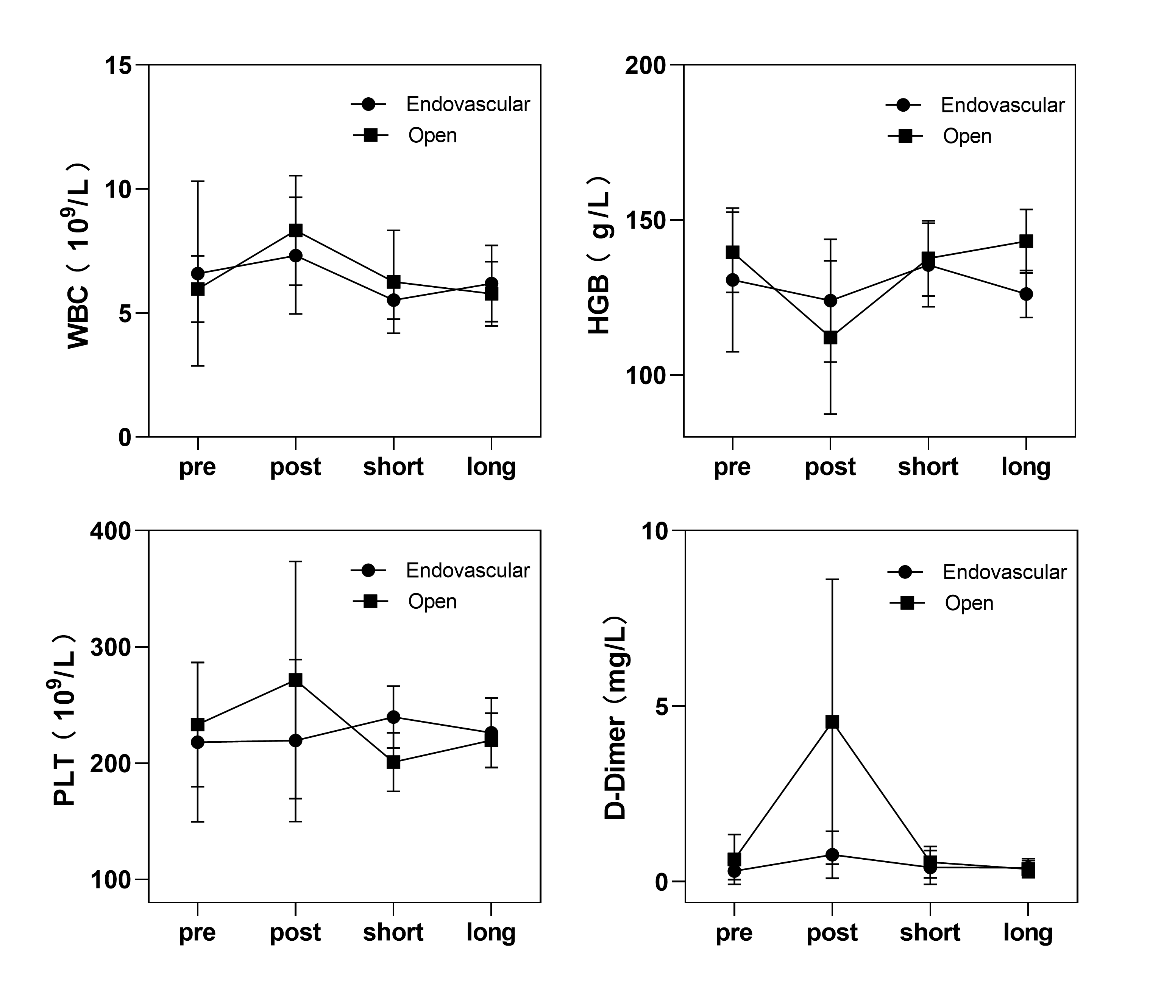


**Supplemental figure 1. Laboratory results at different time-point for RAAs**

Pre-operative, post-operative, short- and long-term laboratory results including white blood cell count (WBC, 10^9^/L), hemoglobin (HGB, g/L), platelet (PLT 10^9^/L), and D-Dimer (mg/L) was summarized.

**Supplemental table 1: Indication for intervention and methods of renal artery reconstruction.**

|  | No. (%) |
| --- | --- |
| Indication for intervention |  |
| Diameter > 2cm | 32 (54.2) |
| Symptoms | 22 (37.3) |
| Rapid growing | 13 (22.0) |
| Refractory hypertension | 3 (5.1) |
| Operation methods |  |
| Endovascular |  |
| Primary coil embolization | 34 (57.6) |
| Stent assisted coiling | 3 (5.1) |
| Coiling + stenting | 3 (5.1) |
| Simple stenting | 5 (8.5) |
| Surgery |  |
| Tailoring | 2 (3.4) |
| Resection and primary anastomosis | 1 (1.7) |
| Resection and saphenous vein graft |  |
| Autogenous reconstruction | 3 (5.1) |
| Ex vivo reconstruction and auto-transplantation | 6 (10.2) |
| Resection and prosthetic graft | 1 (1.7) |
| Clipping | 1 (1.7) |

**Supplemental table 2: Thirty-day and long-term results after reconstruction of RAAs with open surgery or endovascular repair.**

|  | Endovascular (n=45) | Open (n = 14) | *P* |
| --- | --- | --- | --- |
| Thirty-day outcome (%) |  |  |  |
| Technical primary success | 45 (100) | 14 (100) | 1.000 |
| Transient postoperative complication* | 8 (17.8) | 6 (42.9) | 0.075 |
| Long-term outcome (%) |  |  |  |
| Median follow-up, Months [IQR] | 25.1 [33.1] | 64.5 [65.2] | **0.027** |
| Occluded renal arteries or stent/graft | 2 (4.4) | 2 (14.3) | 0.250 |
| Re-intervention | 5 (11.1) | 2 (14.3) | 0.620 |

*Including transient flank pain, fever, and abdominal pain or discomfort. IQR: interquartile range.
